# IE-Vnet: Deep Learning-Based Segmentation of the Inner Ear's Total Fluid Space

**DOI:** 10.3389/fneur.2022.663200

**Published:** 2022-05-11

**Authors:** Seyed-Ahmad Ahmadi, Johann Frei, Gerome Vivar, Marianne Dieterich, Valerie Kirsch

**Affiliations:** ^1^German Center for Vertigo and Balance Disorders, University Hospital, Ludwig-Maximilians-Universität, Munich, Germany; ^2^Department of Neurology, University Hospital, Ludwig-Maximilians-Universität, Munich, Germany; ^3^NVIDIA GmbH, Munich, Germany; ^4^IT-Infrastructure for Translational Medical Research, University of Augsburg, Augsburg, Germany; ^5^Computer Aided Medical Procedures (CAMP), Technical University of Munich (TUM), Munich, Germany; ^6^Graduate School of Systemic Neuroscience (GSN), Ludwig-Maximilians-Universität, Munich, Germany; ^7^Munich Cluster for Systems Neurology (SyNergy), Munich, Germany

**Keywords:** MRI, deep learning, endolymphatic hydros, endolymphatic and perilymphatic space, convolutional neural network CNN, VNet, segmentation (image processing), inner ear imaging

## Abstract

**Background:**

*In-vivo* MR-based high-resolution volumetric quantification methods of the endolymphatic hydrops (ELH) are highly dependent on a reliable segmentation of the inner ear's total fluid space (TFS). This study aimed to develop a novel open-source inner ear TFS segmentation approach using a dedicated deep learning (DL) model.

**Methods:**

The model was based on a V-Net architecture (IE-Vnet) and a multivariate (MR scans: T1, T2, FLAIR, SPACE) training dataset (D1, 179 consecutive patients with peripheral vestibulocochlear syndromes). Ground-truth TFS masks were generated in a semi-manual, atlas-assisted approach. IE-Vnet model segmentation performance, generalizability, and robustness to domain shift were evaluated on four heterogenous test datasets (D2-D5, *n* = 4 × 20 ears).

**Results:**

The IE-Vnet model predicted TFS masks with consistently high congruence to the ground-truth in all test datasets (Dice overlap coefficient: 0.9 ± 0.02, Hausdorff maximum surface distance: 0.93 ± 0.71 mm, mean surface distance: 0.022 ± 0.005 mm) without significant difference concerning side (two-sided Wilcoxon signed-rank test, *p*>0.05), or dataset (Kruskal-Wallis test, *p*>0.05; *post-hoc* Mann-Whitney U, FDR-corrected, all *p*>0.2). Prediction took 0.2 s, and was 2,000 times faster than a state-of-the-art atlas-based segmentation method.

**Conclusion:**

IE-Vnet TFS segmentation demonstrated high accuracy, robustness toward domain shift, and rapid prediction times. Its output works seamlessly with a previously published open-source pipeline for automatic ELS segmentation. IE-Vnet could serve as a core tool for high-volume trans-institutional studies of the inner ear. Code and pre-trained models are available free and open-source under https://github.com/pydsgz/IEVNet.

## 1. Introduction

*In-vivo* non-invasive verification of endolymphatic hydrops (ELH) via intravenous delayed gadolinium (Gd) enhanced magnetic resonance imaging of the inner ear (iMRI) is increasingly becoming an essential standard clinical diagnostic tool to distinguish leading causes of peripheral vestibulocochlear syndromes (1, 2). In this context, a fast and easily reproducible, yet more importantly, comparable and standardized quantification method of the endolymphatic space (ELS) is a prerequisite in any setting, be it clinical or research (3). Unfortunately, such a quantification method is not entirely available yet despite many efforts.

At first glance, clinical radiology approaches offer fast and easily applicable visual semi-quantitative (SQ) ELH classifications (4–9). Nevertheless, given the plurality of visual SQ ELH classification approaches that may vary in wording, resolution (3- or 4-point ordinal scale), or evaluation level (anatomical fixpoint), and can be sensitive to human bias, published results cannot be considered inherently reproducible, comparable or standardized (10). Already an improvement in comparability, manual measurement of the ELS area in 2D within one MR-layer (11, 12), or better yet, the entire ELS volume 3D over multiple MR-layers (13, 14) remain dependent on human decisions.

Similar to optimizing entire iMR sequences in use to date (15–17), automatic ELS quantification is predetermined by two methodical sticking points (18): The first obstacle is to distinguish between total fluid space (TFS) within the entire inner ears bony labyrinth from the surrounding petrosal bone structures (19–21). The second difficulty is distinguishing the two different fluid spaces within the TFS (22, 23), namely ELS within the membranous labyrinth and the surrounding perilymphatic space (PLS) within the bony labyrinth. Current semi-automatic (24–26) or automatic (27, 28) 3D ELS quantification methods have mostly concentrated on ELS differentiation within TFS.

Most available 3D TFS segmentation approaches are either manual (24, 26), or atlas-based (29, 30). However, atlas-based segmentation uses deformable image registration that entails several challenges (31). On the one hand, careful parameterization and run-times between minutes to hours of computation to obtain accurate segmentation prohibit interactive analysis. Another challenge and important motivation for this study are that the thin structures of the TFS, particularly the semi-circular canals, often lead to misregistration, despite the usage of multi-resolution registration.

A promising alternative tool is machine learning algorithms based on deep neural networks (DNN, or deep learning). Recently, an automated 2D measurement of hydrops ratio using a three-layer convolutional neural network (CNN) based segmentation (32) and a deep learning algorithm for fully automated 3D segmentation of the inner ear (33) were proposed. However, to the best of our knowledge, these algorithms are not accessible to the public at large.

This work proposes an open-source approach for inner ear TFS segmentation based on deep learning and using a specialized V-Net architecture (IE-Vnet) that will be made available to the scientific community. The discussion includes a comprehensive comparison of the currently available deep learning algorithms for 3D volumetric inner ear segmentation. In addition, we aimed to investigate the following questions:

(i) Is the training of the IE-Vnet on semi-manual, atlas-based pre-segmentations of inner ear TFS possible from a large cohort with comparatively little manual segmentation effort?
(ii) Is the IE-Vnet able to generalize across domain shift differences in MRI scanner hardware and sequence settings, or patient pathology without significant loss of segmentation accuracy, given appropriate augmentation techniques during training?

## 2. Materials and Methods

### 2.1. Setting and Institutional Review Board Approval

This work was conducted at the interdisciplinary German Center for Vertigo and Balance Disorders (DSGZ) and the Neurology Department of the Munich University Hospital (LMU) between 2015 and 2019. This study used previously published datasets (10, 27, 30, 34, 35). Institutional Review Board approval was obtained before the initiation of the study (no. 094-10 and no. 641-15). All participants provided informed oral and written consent in accordance with the Declaration of Helsinki before inclusion in the study. The inclusion criterion was age between 18 and 80 years. The exclusion criteria were other (than vestibular) neurological or psychiatric disorders, as well as any MR-related contraindications (36), poor image quality, or missing MR sequences.

### 2.2. Datasets and Cohorts

The study included five different real-life datasets, denoted as D1–D5. Dataset 1 (D1, training dataset) was used to train the deep neural network model. Datasets 2–5 (D2–D5, test datasets) were used to investigate the model's out-of-sample performance due to MR scanner, MR sequence, or cohort and pathology. A detailed description of the domain differences between D1 and D2-5 is given in Table 1.

**Table 1:** Domain differences between training (D1) and test (D2-D5) datasets.

	**MR scanner**	**# Channels**	**ELH**	**Vestibulocochlear syndrome**	**Domain difference**
D1	Skyra	20	Yes/No	Yes	No
D2	Skyra	20	No	No	ELH, pathology
D3	Skyra	20	Yes	Yes	No
D4	Verio	32	Unknown, but improbable	No	Scanner, coil, site, ELH, pathology
D5	Verio	32	Unknown, but possible	Yes	Scanner, coil, site

*Test datasets with various properties were included to examine the robustness of the network's segmentation performance toward domain shift. This shift was caused either by changes in population (endolymphatic hydrops present or not, determined by an ELH grade ≥1; pathologies present or not), or by changes in the imaging hardware and sequence parameters (scanner model, number of channels in the head RF coil), or both*.

#### 2.2.1. Training Dataset D1

D1 included 358 ears of 179 consecutive patients (102 female= 56.9%; aged 19–80 years, mean age 52.2 ± 15.7 years) with peripheral vestibulocochlear syndromes that underwent iMRI for exclusion or verification of ELH (51 without ELH, 49 with unilateral ELH, 79 with bilateral ELH). Vestibulocochlear syndromes comprised Meniere's disease (*n* = 78), vestibular migraine (*n* = 69), acute unilateral vestibulopathy (*n* = 14), vestibular paroxysmia (*n* = 11), bilateral vestibulopathy (*n* = 5), and benign paroxysmal positional vertigo (*n* = 2). Patients were clinically diagnosed according to the respective international guidelines, such as the brny Society (www.jvr_web.org/ICVD.html or https://www.baranysociety.nl) when diagnosing vestibular migraine (37, 38), Menires disease (39), vestibular paroxysmia (40), bilateral vestibulopathy (41), acute unilateral vestibulopathy/vestibular neuritis (42) and benign paroxysmal positional vertigo (43). A detailed description of the diagnostic work-up of all cohorts can be found in the Supplementary Material.

#### 2.2.2. Test Dataset D2 and D3

In comparison to D1, these test datasets have the same acquisition parameters (D2, D3) but differences in population (D2). **D2** included 20 ears of 10 consecutive Department of Neurology inpatients (7 female= 70%; aged 24–45 years, mean age 33.1 ± 6.7 years) without symptoms or underlying pathologies of the peripheral and central audio-vestibular system that underwent MRI with a contrast agent as part of their diagnostic workup and agreed to undergo iMRI sequences after 4 h without any indication of ELH. Patients were admitted into the clinic due to movement disorders (*n* = 3), epilepsy (*n* = 2), trigeminal neuralgia (*n* = 2), viral meningitis (*n* = 1), subdural hematoma (*n* = 1), and decompensated esophoria (*n* = 1). D2 underwent audio-vestibular testing confirmed the soundness of their peripheral end organs. **D3** included 20 ears of 10 consecutive patients (6 female= 60%; aged 20–58 years, mean age 37.8 ± 13.6 years) with peripheral vestibulocochlear syndromes that underwent iMRI for verification of ELH (7 with unilateral ELH, 3 with bilateral ELH). Pathologies comprehended patients with Meniere's disease (*n* = 3), vestibular migraine (*n* = 3), acute unilateral vestibulopathy (*n* = 2), vestibular paroxysmia (*n* = 1), and bilateral vestibulopathy (*n* = 1).

#### 2.2.3. Test Dataset D4 and D5

In comparison to D1, these datasets differ regarding MR acquisition parameters (D4, D5) and population (D4). **D4** included 20 ears of 10 consecutive healthy controls (HC; 7 female= 70%; aged 25–52 years, mean age 36.6 ± 9.1 years). **D5** included 20 ears of 10 consecutive patients (4 female= 40%; aged 27–44 years, mean age 37.5 ± 5.6 years) with bilateral vestibulopathy. Measured MR sequences in D4 and D5 only distinguished between TFS within the entire inner ears bony labyrinth from the surrounding petrosal bone structure, but not between ELS and PLS within the TFS. The existence of an ELH cannot be excluded, but is unlikely in D4 and possible in D5.

### 2.3. MR Imaging Data Acquisition

#### 2.3.1. Datasets D1-3

Four hours after intravenous injection of a standard dose (0.1 mmol/kg body weight) of Gadobutrol (Gadovist^Ⓡ^, Bayer, Leverkusen, Germany), MR imaging data was acquired in a whole-body 3 Tesla MR scanner (Magnetom Skyra, Siemens Healthcare, Erlangen, Germany) with a 20-channel head coil. Head movements were minimalized in all three axes using a head positioning system for MRI (Crania Adult 01, Pearl Technology AG, Schlieren, Switzerland). A 3D-FLAIR (fluid-attenuated inversion recovery) sequence was used to differentiate ELS from PLS within TFS, and a spin-echo 3D-SPACE (three-dimensional sampling perfection with application-optimized contrasts by using different flip angle evolutions) sequence to delineate the TFS from the surrounding bone. ELH was classified on 3D-FLAIR images as enlarged negative-signal spaces within TFS, according to a previously reported convention (8, 10). The 3D-FLAIR had the following parameters: TE 134 ms, TR 6,000 ms, TI 2240 ms, FA 180°, FOV 160 × 160 *mm*^2^, 36 slices, base resolution 320, averages 1, acceleration factor of 2 using a parallel imaging technique with a generalized auto-calibrating partially parallel acquisition (GRAPPA) algorithm, slice thickness 0.5 mm, 0.5 × 0.5 × 0.5 *mm*^3^ spatial resolution.

The spin-echo 3D-SPACE sequence had the following parameters: TE 133 ms, TR 1000 ms, FA 100°, FOV 192 × 192 *mm*^2^, 56 slices, base resolution 384, averages 4, acceleration factor of 2 using GRAPPA algorithm, 0.5 mm slice thickness, 0.5 × 0.5 × 0.5 *mm*^3^ spatial resolution. Further structural sequences included a T2-weighted sequence (TE 89 ms, TR 4,540 ms, FOV 250 × 250 *mm*^2^, 42 slices, base resolution 364, averages 1, acceleration factor of 2 using GRAPPA algorithm, slice thickness 3 mm, voxel size 0.7 × 0.7 × 3 *mm*^3^) and a T1-weighted magnetization-prepared rapid gradient echo (MP-RAGE) sequence with an isotropic spatial resolution of 1.0 × 1.0 × 1.0 *mm*^3^ (TE 4.37 ms, TR 2,100 ms, FOV 256 × 256 *mm*^2^, 160 slices).

#### 2.3.2. Datasets D4-5

MR imaging data were acquired in a whole-body 3.0 Tesla MR scanner (Magnetom Verio, Siemens Healthcare, Erlangen, Germany) with a 32-channel head coil. Head movements were minimalized in all three axes using a head positioning system for MRI (Crania Adult 01, Pearl Technology AG, Schlieren, Switzerland). A spin-echo 2D-SPACE sequence was used to delineate the bony labyrinth (TR 1,000 ms, TE 138 ms, FA 110°, FOV 180 × 180 *mm*^2^, 60 slices, base resolution 384, averages 2, slice thickness 0.5 mm, 0.5 × 0.5 × 0.5 *mm*^3^ spatial resolution). Further structural sequences included a T2-weighted sequence (TE 94 ms, TR 4,000 ms, FOV 230 × 230 *mm*^2^, 40 slices, base resolution 364, averages 1, acceleration factor 2 using GRAPPA algorithm, slice thickness 3 mm, voxel size 0.7 × 0.7 × 3 *mm*^3^) and a T1-weighted magnetization-prepared rapid gradient echo (MP-RAGE) sequence with a field-of-view of 256 mm and an isotropic spatial resolution of 1.0 × 1.0 × 1.0 *mm*^3^ (TE 4.37 ms, TR 2,100 ms, 160 slices).

### 2.4. Creation of Ground Truth Using Atlas-Based Segmentation

**Figure 1:**
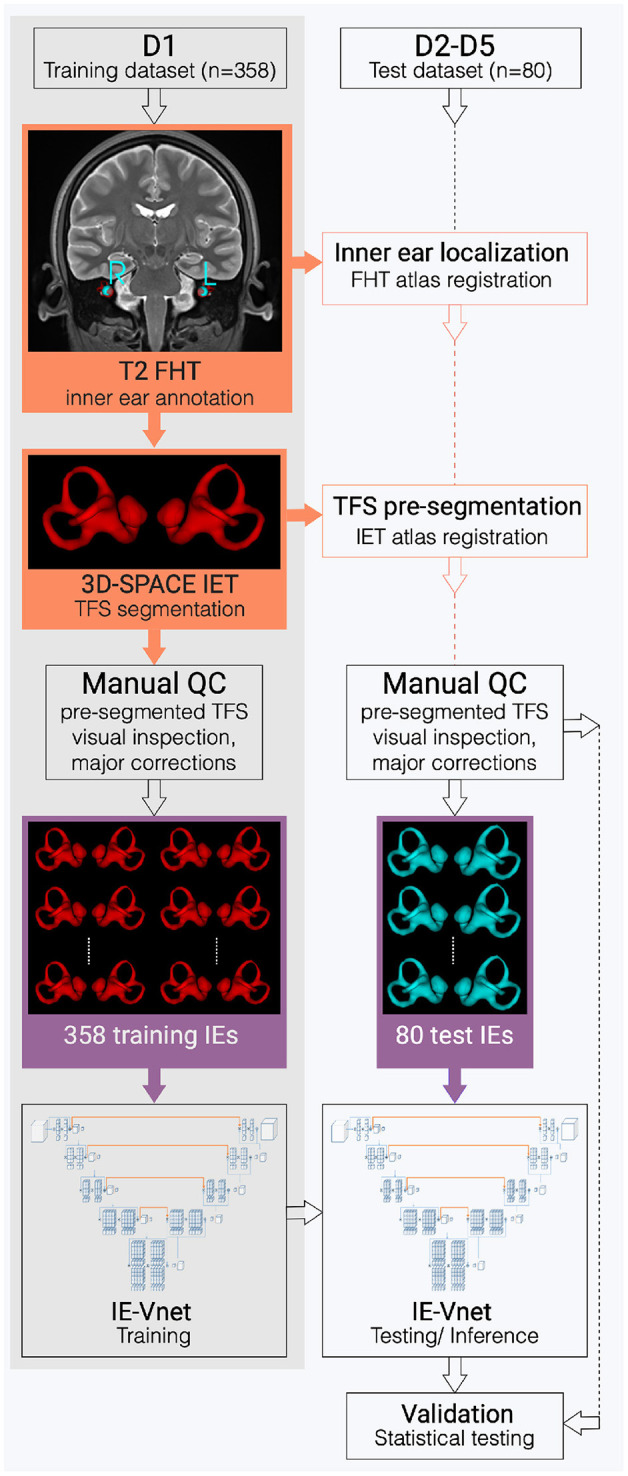
Flowchart of the inner ear's auto-segmentation. Auto-segmentation of the inner ear (IE) involved data preparation and manual ground-truth annotation of the IEs total fluid space (TFS) masks in training (D1, grey shading) and test (D2-D5, white shading) datasets. First, pre-segmentations (orange boxes) were obtained in D1-D5 via a custom-built full-head template (FHT) and an inner-ear template (IET). Then, manual quality control (QC), followed by manual refinement of IE segmentations (purple boxes), trained and examined the IE-Vnet model. Finally, its predictions were validated under various forms of domain shift in the test datasets D2-D5 (cf. Table 1).

**Figure 2:**
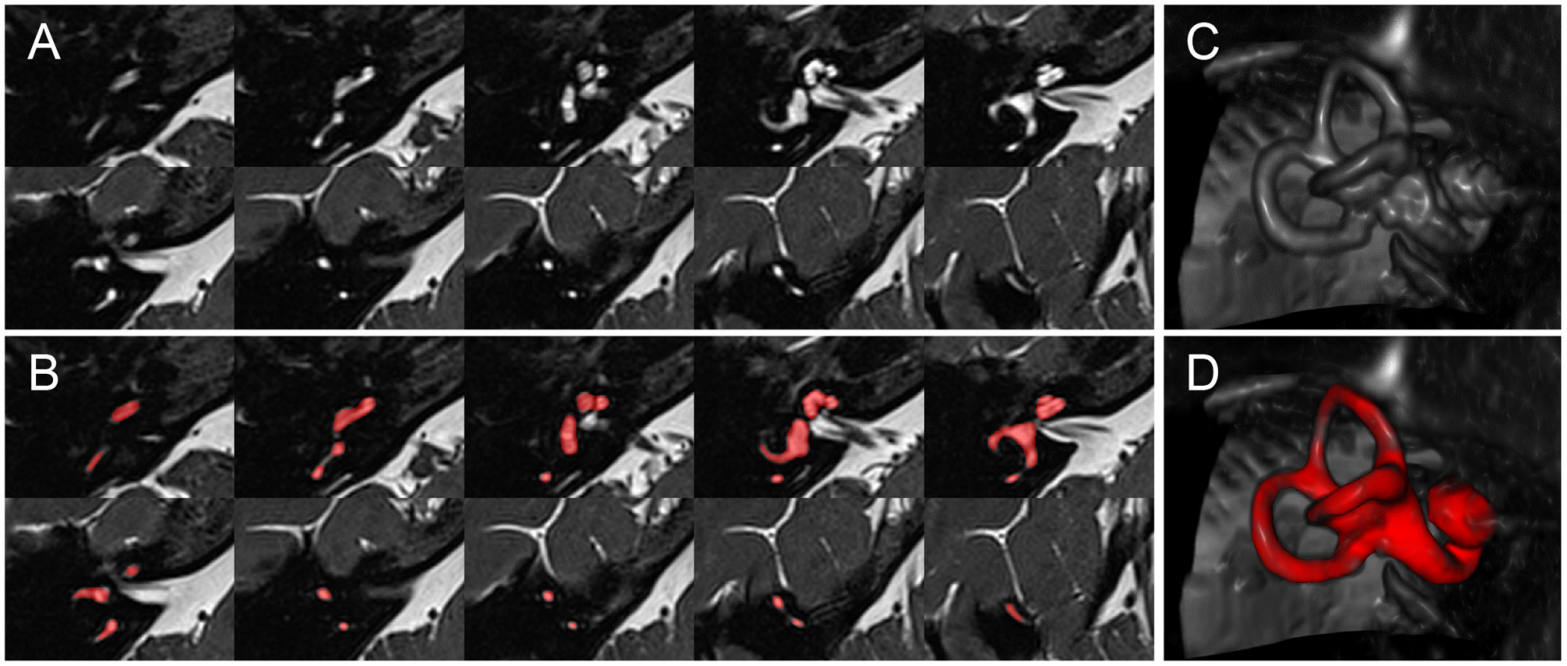
Inner ear MR example case. Depiction of an exemplary SPACE volume along with its ground-truth segmentation masks. **(A)** Depiction of ten axial slices from the SPACE MRI sequence volume, through the right inner ear, from caudal to cranial, covering a range of 12.4 mm (~1.4 mm slice distance). **(B)** Like **(A)**, but with the manually segmented total fluid space (TFS) mask (colored in red). **(C)** Volume rendering of the right inner ear ROI, which serves as input to the IE-Vnet model. **(D)** Like **(C)**, but the manually segmented TFS surface overlaid in red.

The ground-truth (or gold standard) segmentation for D1-5 was created using the T2 and SPACE MRI volumes in a semi-manual process, with the assistance of automatic, atlas-based segmentation. 2D- or 3D-SPACE MRI volumes served as input to the IE-Vnet model. A flowchart of the (semi-)manual ground-truth segmentation can be viewed in Figure 1. Figure 2 depicts an exemplary T2 volume along with a ground-truth segmentation mask. First, two custom templates and atlases were created from scratch, specifically for automated pre-segmentation of the inner ear. Then, registrations were performed using linear affine and non-linear Symmetric Normalization [SyN, (44)] as well as Optimal Template Building [OTB, (45)], which are part of the Advanced Normalization Toolkit (ANTs)^1^. Also, all subjects T1, T2 and FLAIR volumes were spatially co-aligned with the SPACE volume via intra-subject rigid registration.

The first atlas localized the inner ears inside full-head (FH) or limited FOV (field-of-view) MRI scans. To this end, a full-head template (FHT) was created from T2 volumes using ANTs OTB, and the inner ear structures' central location was annotated with a single landmark for each side, respectively. Finally, FHT plus annotations, i.e., the full-head atlas, were non-linearly registered to all subjects' volumes. Thus, left and right inner ears could be located in all participants's heads.

The second atlas enabled automatic pre-segmentation of the inner ear. Therefore, inner ear localization landmarks were transferred from the FH T2-FLAIR scans to the narrow FOV SPACE scans. Here, inner ears were cropped using a 4 × 3 × 2 cm region-of-interest (ROI) that contained the entire inner ear structure and a sufficient margin of 5–10 mm to all sides to account for slight localization errors. Inside the ROI, SPACE voxel intensities were resampled at 0.2 mm isotropic resolution (i.e., 200 × 150 × 100 voxels). All ROI cubes were geometrically centered to the origin ([0, 0, 0]) coordinate. At the origin, right-sided inner ears were re-oriented onto the left inner ears through horizontal flipping. A single inner ear template (IET) using ANTs OTB was computed from this uni-directed set of inner ears. This template was annotated with manual segmentation of the total fluid space (TFS), first by intensity thresholding with Otsu's method (46), followed by manual refinement with various 3D mask editing tools “Segment Editor Module”, mainly 3D brush, eraser, and scissor tool in 3D Slicer ^2^ (47).

All inner ears in training (D1) and testing (D2-5) were pre-segmented using two atlas registrations; first, an inner ear localization with the FHT, followed by TFS segmentation with the IET. Then, an automatic refinement step was performed post-registration by intersecting an Otsu-thresholded mask with a 0.5 mm dilated atlas mask to account for patient-wise shape- and intensity- variations. Despite this automatic refinement, every automatic segmentation needed to be quality-controlled (QC) and corrected for mistakes in an additional manual process. Two different QC and correction strategies were implemented in the training dataset (D1) and test datasets (D2–D5) to balance the amount of manual annotation effort and the TFS masks criticality. The automatic segmentation underwent a visual QC check in each of the 358 training inner ears (D1). Inner ear localization worked very robustly, without any inner ears being missed or mislocalized. In contrast, the atlas-based segmentation was not as robust, with severe mis-segmentations (e.g., partially incomplete or entirely missed semi-circular canals or cochlear turns) in 64 out of 358 training inner ear ROIs (17.9%). These were manually refined before network training, while the remaining 302 inner ears were used for training, even if minor visual errors in the atlas auto-segmentations were present. In contrast, atlas-segmentation in the test datasets (D2–D5) was not only visually inspected, but all 80 inner ears were thoroughly error-corrected and manually refined with the aforementioned 3D Slicer mask editing tools. Manual refinement of a single inner ear, for an experienced annotator familiar with the 3D Slicer user interface, took on the order of 5–15 min.

The pre-processing steps necessary for inner ear segmentation in new MRI volumes are limited to localizing the left and right inner ear. This can be achieved automatically using a full-head registration (performed in this work) and requires no manual interaction. Alternatively, the inner ears can also be manually localized using landmark annotation. Depending on the workstation hardware and registration parametrization, a fully automatic inner ear ROI localization can be performed in 1–2 min. However, a manual localization is much faster and requires two clicks, which can be performed in seconds.

### 2.5. IE-Vnet Neural Network Architecture and Training

#### 2.5.1. Architecture and Loss Function

**Figure 3:**
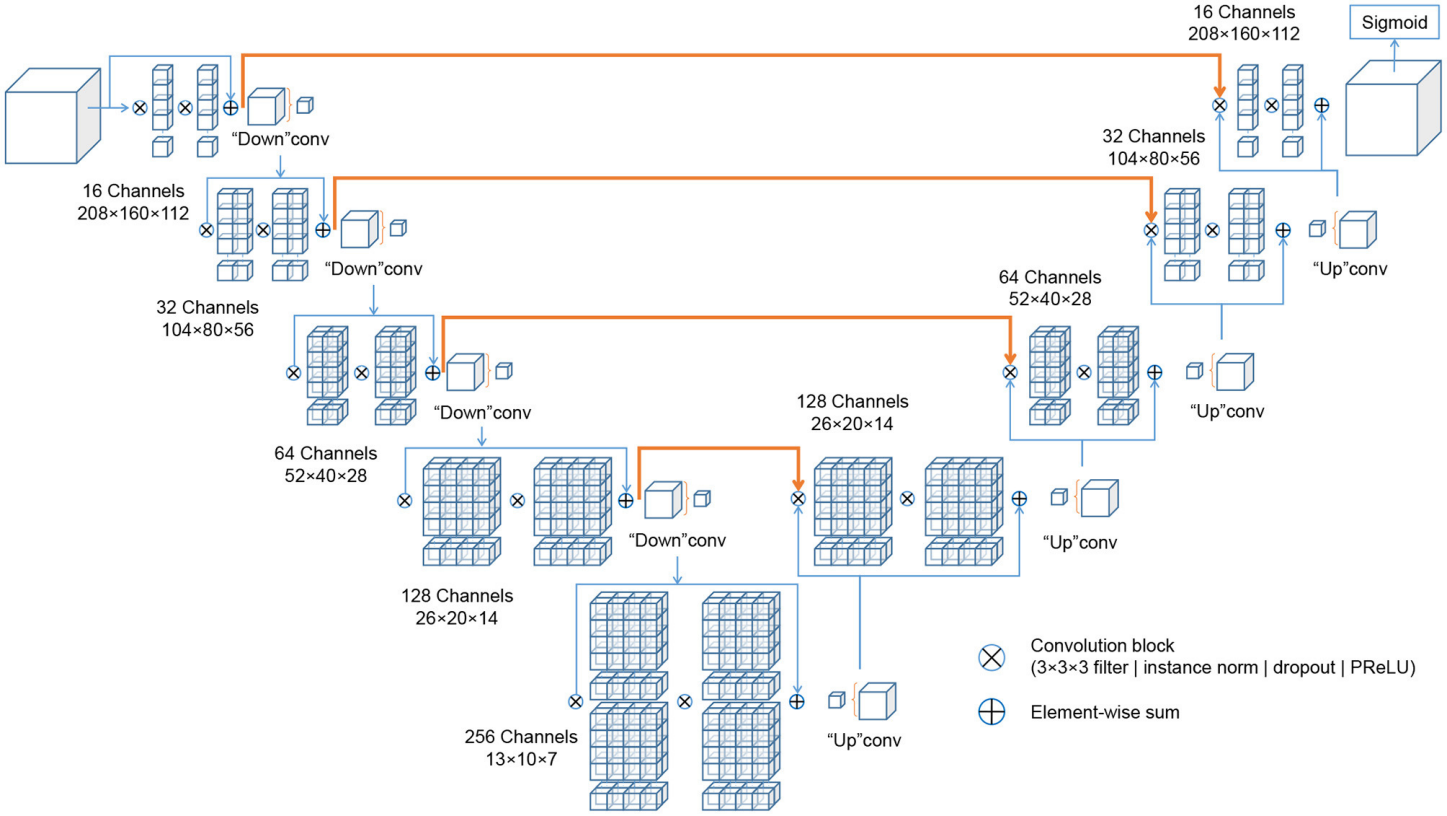
Schematic representation of the IE-Vnet architecture. The IE-Vnet architecture is designed as a variant of a V-Net architecture with four down-sampling levels, [16, 32, 64, 128, 256] 3D-convolutional filters at each level (kernel size: 3 × 3 × 3 voxels), and with residual blocks spreading two convolutional layers each within each level. Each convolutional layer is followed by Instance Normalization (50), channel-wise random dropout (*p* = 0.5), and non-linear activation with Parametrized Rectified Linear Units (PReLU) (51). The convolutions aim to extract features from the data and, at the end of each stage, reduce its resolution by using appropriate stride. The left part of the network consists of a compression path, while the right part decompresses the signal until its original size is reached. A more detailed description can be found in Milletari et al. (48).

The deep learning architectures for volumetric 3D segmentation were based on a V-Net model (48), which is a variant of the 3D U-Net family of architectures (49). The basic idea of these fully convolutional architectures is to extract hierarchical image features using learnable convolutional filters at an increasingly coarse resolution and image representation. The down-sampling and up-sampling operations are achieved via pooling/un-pooling operations (49) or forward/transpose convolutions (48). In this work, the network was designed as a variant of a V-Net architecture, with four down-sampling levels, with [16, 32, 64, 128, 256] 3D-convolutional filters at each level (kernel size: 3 × 3 × 3 voxels), and with residual blocks spreading two convolutional layers each within each level. Each convolutional layer is followed by Instance Normalization (50), channel-wise random dropout (*p* = 0.5), and non-linear activation with Parametrized Rectified Linear Units (PReLU) (51). The loss function used for training was the Dice loss (48). The recently published cross-institutional and open-source deep learning framework “Medical Open Network for AI” (MONAI) (52)^3^ was used to implement the network, pre-processing, augmentation and optimization. Figure 3 visualizes the architecture.

#### 2.5.2. Pre-processing and Augmentation Scheme

All volumes in D1–D5 were pre-processed with simple spatial padding to a volume size [208, 160, 112], and intensity scaling to the range [0…1]. The dataset D1 was split into 90% training data (*N* = 161 subjects, 322 inner ears) and 10% validation data (*N* = 18 subjects, 36 inner ears). Random image augmentation was used to enlarge the training set size artificially, since fully convolutional segmentation networks require large amounts of training data for robust and accurate prediction. Augmentation steps included random contrast adjustment (gamma range: [0.3, …1.5], probability of occurrence *p*_*o*_ = 0.9), addition of random Gaussian noise (μ = 0, σ = 1.0, *p*_*o*_ = 0.5), random horizontal flipping (*p*_*o*_ = 0.5), and random affine-elastic transformation (*p*_*o*_ = 0.75; 3D translation: 15% of ROI dimensions; 3D rotation: 20°; scaling: ±15%; grid deformation: magnitude range [5…100], sigma range: [5…8]).

#### 2.5.3. Optimization

Adam stochastic optimization algorithm (53) at a learning rate of 3*e*−4 was used to train the network weights.

### 2.6. Validation Parameters

Segmentation accuracy was quantified using spatial overlap indexes, such as Dice overlap coefficient (54, 55), Hausdorff distance (56, 57), and mean surface distance (58).

Localized performance issues within the inner ear were visually assessed using a semi-quantitative five-point Likert-type response scale (59, 60). Therefore, the level of agreement in the segmentation outcome of the cochlea, sacculus, utriculus, the anterior semi-circular canal (aSCC), posterior SCC (pSCC), and horizontal SCC (hSCC), respectively, were quantified using the following categories: 5-Strongly agree (no structure missing, no false-positive segmentation, clean contour), 4-Agree (no structure missing, no false-positive segmentation, ≤ 1 unclean contour), 3-Neither agree nor disagree, (no structure missing, ≤ 1 false-positive segmentation, > 1 unclean contour), 2-Disagree ( ≤ 1 missing structure, > 1 false-positive segmentation, clean or unclean contour), and 1-Strongly disagree (> 1 missing structure, > 1 false-positive segmentation, clean or unclean contour).

### 2.7. Statistical Testing

Normal distribution of Dice overlap measures across datasets was determined using Shapiro and Wilk testing (61) and homoskedastic across datasets was determined using Bartlett and Fowler testing (62) before statistical analysis. Consequently non-parametric testing was further applied. Given their ordinal nature (63), non-parametric testing was also applied to the Likert-type expert ratings.

Statistical hypothesis tests were then performed to investigate two questions: First, the sidedness of the network was checked, i.e., whether there was a statistically significant difference in segmentation accuracy (Dice overlap coefficients, Likert ratings) between left and right inner ears. To this end, a non-parametric Wilcoxon signed-rank test was applied to the Dice, and Likert outcomes, paired between the left and right inner ears of each test subject. Second, the null-hypothesis was verified, i.e., that the Dice overlap median outcomes of the four test datasets D2-D5 were equal. The purpose of this was to investigate the generalization capability of the network, i.e., whether a shift in population or imaging parameters or both (cf. Table 1) led to a measurable deterioration of segmentation performance. To this end, a non-parametric Kruskal-Wallis test for independent samples was employed with the concatenated left and right Dice and Likert outcomes as the dependent variable and the test set indicator (D2–D5) as the independent variable. *Post-hoc*, a non-parametric tests [Mann-Whitney U (64)] between Dice and Likert outcomes was performed in all pairs of test datasets D2–D5. All statistical analyses were applied using the open-source libraries Scipy Stats (65), Statsmodels (66), and Pingouin (67). Values are presented as means ± standard deviations.

## 3. Results

Results are presented separately for the training and testing stage, followed by statistical comparisons.

### 3.1. Training Results

**Figure 4:**
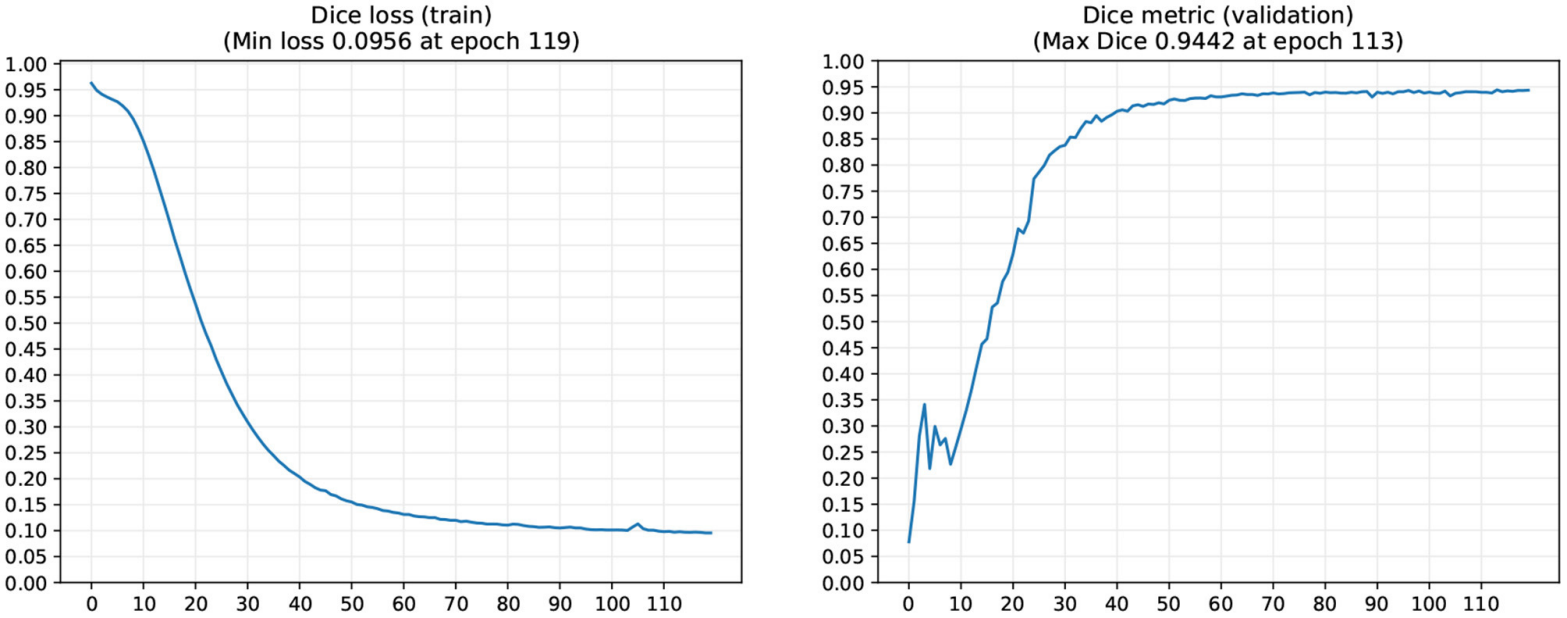
IE-Vnet training loss and validation metrics. IE-Vnet training loss **(left graphic)** and validation **(right graphic)** metrics were observed during 120 epochs of training. In addition, the maximum validation Dice overlap metric of 0.944 was obtained at epoch 113. Notably, the loss curve showed a steady convergence toward the minimum obtained at the final iteration. At the same time, the validation metric showed a steady convergence without any signs of overfitting throughout the entire optimization procedure.

Figure 4 shows the evolution of Dice loss for model training and the corresponding Dice metric on the withheld validation set. The maximum validation Dice overlap metric of 0.944 was obtained at epoch 113, and this best-performing model was saved for forwarding inference on the withheld test datasets D2-D5 (cf. Sections 3.2, 3.3), as well as for open-source dissemination. Notably, the loss curve showed a steady convergence toward the minimum obtained at the final iteration. Simultaneously, the validation metric showed a steady convergence without any signs of overfitting throughout the entire optimization procedure. The total training time took around 11 h on a consumer-level workstation (AMD Ryzen Threadripper 1950X 8-core CPU, 32 GB RAM, Nvidia 1080 Ti GPU).

### 3.2. Test Results

**Figure 5:**
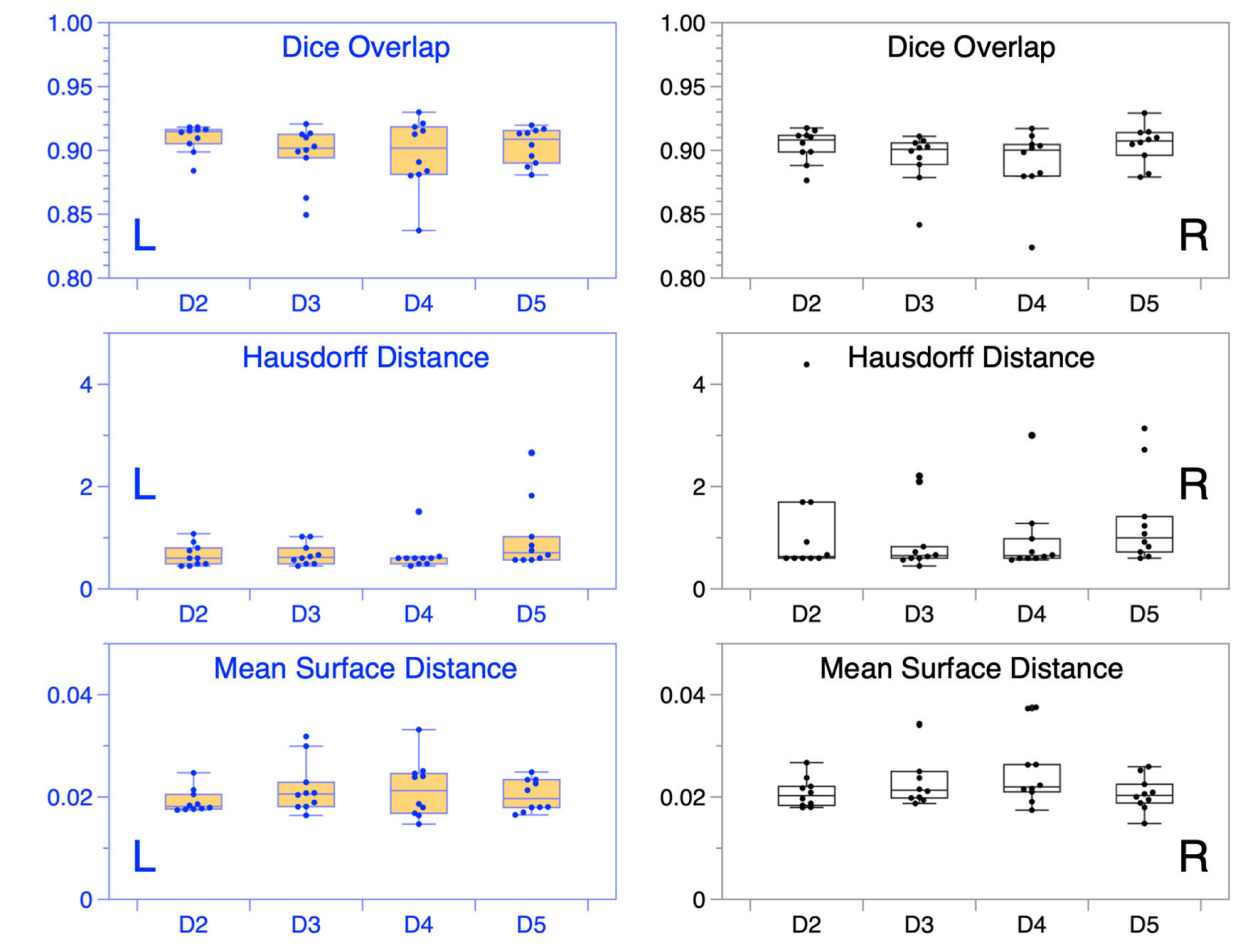
IE-Vnet segmentation quality control. Alignment between ground-truth and prediction in the four test datasets D2-D5 (20 IE each, 80 IE altogether) was measured by the quantitative metrics of Dice overlap coefficient **(upper row)**, Hausdorff maximum surface distance **(middle row)**, and average surface distance **(lower row)**. Results of left ears (blue) are depicted on the left (L), while results of the right ears (black) are shown on the right (R). In most cases across D2-5, congruence between model prediction and manual ground-truth was high.

The total inference time for 80 samples was 15.2 s, i.e., on average 0.19 s ± 0.047 s for each cropped and up-sampled inner ear volume at 0.2 mm isotropic resolution (i.e., 200 × 150 × 100 voxels). The agreement of TFS segmentation between manual ground-truth and the networks prediction was quantified by three metrics: Dice overlap coefficient “Dice”, maximum Hausdorff surface distance “HDmax”, mean surface distance “SDmean”, along with five-point Likert-type response scale “LS”. These metrics are illustrated with boxplots in Figure 5, and summarized numerically in Table 2.

**Table 2:** IE-Vnet segmentation results on four test datasets D2-D5 (20 inner ears each) compared to manual ground-truth.

**Dataset**	**D2**		**D3**		**D4**		**D5**	
	**Mean**	**SD**	**Mean**	**SD**	**Mean**	**SD**	**Mean**	**SD**
**(A) Accuracy**								
Dice	0.906	0.012	0.895	0.021	0.904	0.014	0.894	0.026
HDmax	0.949	0.862	0.804	0.476	1.170	0.774	0.811	0.565
SDmean	0.020	0.003	0.023	0.005	0.021	0.003	0.023	0.006
**(B) Performance**								
Cochlea	4.950	0.224	4.900	0.308	4.900	0.308	0.950	0.224
Sacculus	5.000	0.000	5.000	0.000	4.950	0.224	5.000	0.000
Utriculus	5.000	0.000	5.000	0.000	5.000	0.000	5.000	0.000
aSCC	4.800	0.616	4.800	0.616	4.900	0.308	4.750	0.716
pSCC	4.900	0.447	4.900	0.447	4.850	0.671	4.900	0.308
hSCC	4.950	0.224	4.850	0.671	4.850	0.671	4.800	0.696

*Segmentation accuracy (A) was measured by the quantitative metrics of Dice overlap coefficient (“Dice”), Hausdorff maximum surface distance (“HDmax,” in [mm]), and average surface distance (“SDmean,” in [mm]). Localized performance (B) issues within the inner ear were assessed using a semi-quantitative five-point Likert-type response scale for the cochlea, sacculus, utriculus, the anterior semi-circular canal (aSCC), posterior SCC (pSCC), and horizontal SCC (hSCC) respectively. A detailed description of the used categories can be found in Section 2.6*.

**Figure 6:**
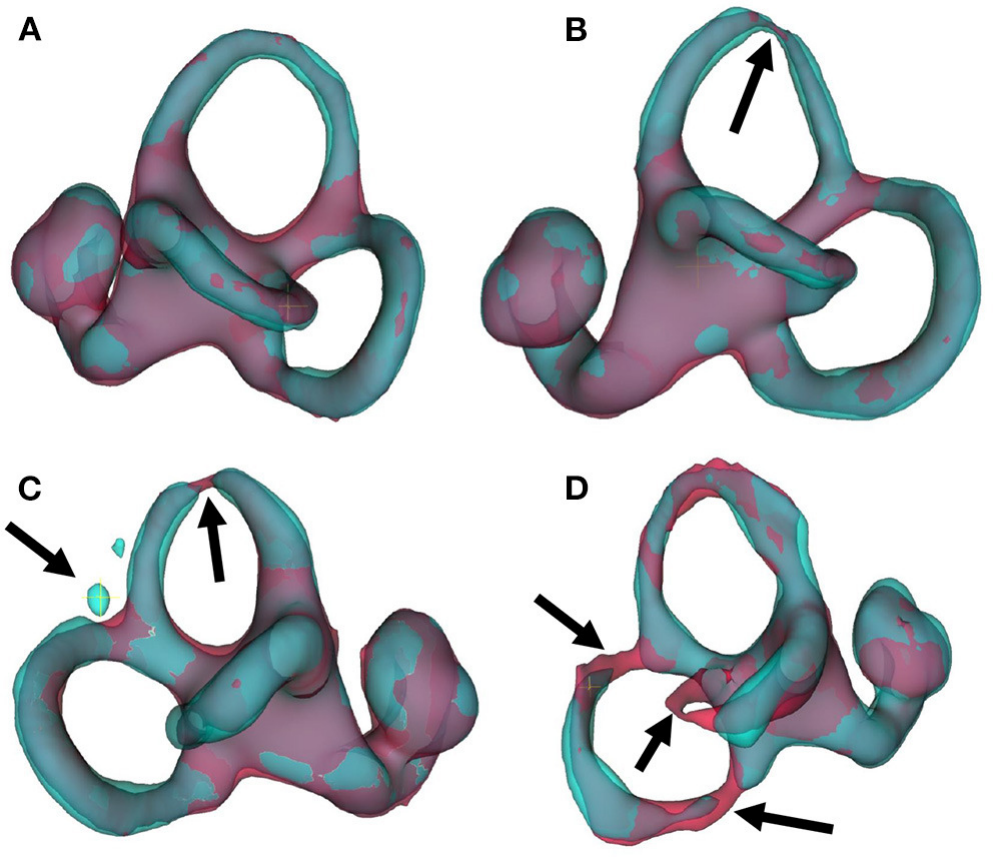
IE-Vnet 3D segmentation predictions visualized. Visualization of 3D segmentations predicted by the IE-Vnet model (color: cyan), overlaid onto manual ground-truth 3D surface contours (color: red). **(A)** Exemplary case of a highly accurate segmentation Dice >0.92, Hausdorff maximum surface distance <0.5 mm, mean surface distance <0.02 mm, 5/5 Likert scale (LS) for cochlea, sacculus, utriculus, anterior, posterior and horizontal SCC. Manual ground-truth and prediction are in high agreement over the entire surface. **(B)** Another example of a highly accurate segmentation. The arrow denotes a location with hypo-intense semi-circular canal voxels, as marked manually in red, and the model prediction predicts a thinning at the same location. LS was 5/5 for cochlea, sacculus, utriculus, anterior, posterior and horizontal SCC. **(C)** An exemplary case of an overall accurate segmentation, but with a high Hausdorff surface distance of >4 mm. LS was 5/5 for cochlea, sacculus, utriculus, posterior and horizontal SCC, and 2/5 for anterior SCC. Visual inspection reveals two isolated blobs, which could be removed with simple post-processing, such as connected components analysis and removal of small islands. **(D)** A failure case. Notably, manual ground-truth creation in this sample was challenging as well. LS was 5/5 for utriculus and anterior SCC, 4/5 for cochlea and sacculus, 2/5 for horizontal SCC, and 1/5 for posterior SCC. Low-contrast and high noise in SPACE sequences, with further potential challenges like motion artifacts, can lead to irregular ground-truth contours (left arrow) or unusually thin semi-circular canal segments (middle and right arrow). In such cases, model predictions may result in in-contiguous semi-circular canals and noisy surface contours.

Several points are noteworthy. On average, across all left and right inner ears and in all four test datasets, the Dice overlap coefficient showed a mean value of 0.900 ± 0.020, the Hausdorff maximum surface distance a mean value of 0.93 ± 0.71 mm), and the mean surface distance a mean value of 0.022 ± 0.005 mm). Thus, the segmentation performance seems quantitatively consistent across the test datasets D2–D5 (cf. Figure 5 and Table 2A), which was further confirmed by statistical analyses (cf. Section 3.3). The mean Likert scales of the inner ear structures were altogether consistently high (4.913 ± 0.337) across both inner ears and in all four test datasets. However, depending on the location, shape and intricacy of the separate inner ear structures, Likert scores consistently differed in performance success (cf. Table 2B) with the most robust results in the vestibulum (sacculus: 4.988 ± 0.112, utriculus: 5.000 ± 0.000), intermediate results in cochlea (4.925 ± 0.265) and posterior SCC (4.888 ± 0.477), and least robust results in the anterior (4.813 ± 0.576) and horizontal SCC (4.863 ± 0.590). The mentioned pattern can be verified in the several outliers, in particular in the Hausdorff distance values in both right and left inner ears. Two cases with outlier Hausdorff distances on the order on 3 mm and above are presented in Figures 6C,D. Visual inspection reveals that these comparatively high surface errors stem either from challenging cases, which were also difficult in manual ground truth segmentation in the horizontal and posterior SCC (panel D), or minor prediction artifacts in the anterior SCC such as isolated blobs, rather than gross mis-segmentations (panel C). Such artifacts could be filtered away through minor post-processing like connected-components filters. In most cases, it is noteworthy that surface congruence between model prediction and manual ground truth was very high, with mean surface distances on the order on 0.02 mm, and with very few cases of surface distances above 0.03 mm. This is also reflected in the form of visual agreement between ground truth and prediction, as visible in two cases in Figures 6A,B.

### 3.3. Impact of Side and Domain Shift

We investigated whether the IE-Vnet segmentation model is affected by a side bias and whether its segmentation performance is affected by variance of the population or imaging parameters (cf. Table 1). The first test was performed in a paired manner between Dice overlap measures in the left and right inner ears for all 40 test subjects (D2–D5). This analysis yielded no significant difference between sides (two-sided Wilcoxon signed-rank test: *p* = 0.061; normality rejected, Shapiro-Wilk test: *p* < 0.001). Further, we examined whether differences in image acquisition led to domain shifts across the four test datasets that impacted our model's segmentation performance. This test yielded no significant difference between Dice overlap outcomes across datasets D2–D5 (Kruskal-Wallis test: *p* = 0.146; homoscedasticity rejected, Bartlett test: *p* < 0.01). Further, the pair-wise *post-hoc* tests between datasets D2-D5 yielded no significant differences in Dice overlaps (Mann-Whitney U, all BH-FDR corrected *p*-values at *p*>0.20). Equivalent results were obtained for qualitative expert ratings of segmentation results upon visual inspection. No significant differences in Likert scale ratings were found across any of the rated regions (cochlea, sacculus, utriculus, anterior, posterior, and horizontal SCC), neither between sides (Wilcoxon signed-rank test, all *p*-values above *p* = 0.162), nor between group-medians across D2–D5 (Kruskal-Wallis test: all *p*-values above *p* = 0.392), nor pair-wise across D2–D5 (*post-hoc*, Mann-Whitney U, all BH-FDR corrected *p*-values at *p*>0.860).

## 4. Discussion

The current work proposes a novel inner ear TFS segmentation approach using a dedicated deep learning (DL) model based on a V-Net architecture (IE-Vnet). A variant of a V-Net deep convolutional neural network architecture was trained to perform segmentation inference on inner ear volumes. During training, various image augmentation techniques were used to account for expected variations in out-of-sample datasets, such as image contrast and intensity, noise, or affine/deformable distortions of geometry. The training dataset was constructed through atlas-based pre-segmentations with comparatively minor manual correction and segmentation effort (aim i). As a result, the inferred IE-Vnet segmentations on four testing datasets were free from side bias and robust to various domain shift sources, such as MRI scanner hardware and sequences and patient pathology (aim ii). Compared to atlas-based segmentation, the novel model was roughly 2,000 times faster and managed to avoid gross mis-segmentations in more than 20% of test cases, especially in high-volume datasets. In the following, IE-Vnet, compared to currently available neural network algorithms used for MR inner ear segmentation, its technical and clinical implications, methodical limitations, and future work will be discussed.

### 4.1. Technical Implications

#### 4.1.1. Accuracy of Segmentation

The average Dice values during testing (0.900) are noticeably lower when compared to training (0.944). This effect can be attributed to the fact that the TFS ground-truth regions were manually refined in every test sample with considerably more effort than the training set. Nevertheless, these indicate accurate segmentation (31), especially in structures like the semi-circular canals, where the Dice metric is known to degrade quickly for small regions or regions with fine-grained protrusions (68). Furthermore, the overall low surface distance of 0.02 mm can be attributed to the fact that the volumes were bi-cubically up-sampled to a resolution of 0.2 mm before inference. Therefore, the manual ground-truths predicted outer surfaces are smooth and consistent even in the presence of fine-grained details.

#### 4.1.2. Generalization

The validation metric showed a steady convergence without any signs of overfitting throughout the entire optimization procedure that points to a well-parameterized network and data augmentation scheme. Furthermore, the results from our statistical analyses on Dice overlap in both inner ears imply the models freedom from side bias. Further, Dice overlap comparisons (group- and pair-wise) across the four testing datasets show no measurable difference in segmentation performance, indicating that the trained network is robust to variations in scanner hardware, image sequence parameters, and population characteristics. When discussing generalization, it is important to also consider whether quantitative metrics are sufficient to obtain trustworthy and interpretable results. A recent study (69) on chest X-ray classification for computer-aided diagnosis of COVID-19 cases has shown that it is vital to incorporate expert validation into the validation of results. Otherwise, it is possible that AI models learn to classify disease statuses based on confounding factors, rather than based on true pathology image content. In particular, image segmentation suffers less from the danger of spurious correlations than image classification: the segmentation output can be overlaid with the source image, and the model predictions become inherently interpretable. However, apart from quantitative metrics like Dice overlap score, or Hausdorff surface distance, a model validation can benefit from additional, expert-based qualitative ratings of the segmentation result. Hence, a differentiated Likert scale rating for the different inner ear structures (cochlea, sacculus, utriculus, anterior, posterior and horizontal semi-circular canal) was incorporated and obtained further insight into the model's performance. In particular, a performance pattern became evident in which, in decreasing order, the most robust results were found in the vestibulum (sacculus, utriculus), while cochlea and posterior SCC performed moderately well. Horizontal SCC and anterior SCC were most susceptible to segmentation errors. Notably, the lack of statistically significant differences in Likert ratings confirms that our model generalizes well. Ideally, these results should be corroborated in further prospective studies and larger cohorts.

#### 4.1.3. Inference Speed Compared to Atlas

On average, the segmentation of a single volume with IE-Vnet took 0.19 ± 0.047 s, including volume loading and pre-processing, and 0.093 s for inference alone. The average segmentation time for inner ears was 377.0 ± 36.9 s using deformable registration. In total, the segmentation was about 2,000 times faster than a state-of-the-art atlas-based method. However, atlas registration is computed on the CPU, while the inference is fully GPU accelerated; hence the comparison is not entirely fair. It is worth noting that GPU-accelerated deformable registration libraries were introduced recently with speedups in the order of 10–100 times (70). Moreover, deep models for deformable (71) and diffeomorphic (72) image registration were recently proposed, allowing for registration times comparable to those of our model. However, deep models for registration are trained with dataset sizes in the order of a few thousand sample volumes (71, 72). Furthermore, atlas-based registration was less robust than IE-Vnet segmentation, as all test dataset volumes required manual correction after atlas pre-segmentation. Hence, our IE-Vnet model was not only trained on TFS contours obtained from registration. Instead, our segmentation model learned patient-wise adaptations, including individual threshold-based refinements and entire manual corrections of atlas auto-segmentations. Patient-specific prediction of the TFS contour, along with the fast inference in the order of milliseconds, makes deep convolutional network models like IE-Vnet attractive for large-scale studies in clinical and neuroscientific imaging-based studies of the inner ear.

#### 4.1.4. Robustness Compared to Atlas

Atlas-based auto-segmentation in our datasets led to severe mis-segmentations (e.g., incomplete or missing semi-circular canals or cochlear turns), which occurred in 17.9% of cases in the training dataset (D1) and 22% of all cases in the test datasets (D2-5), and almost all cases in D2-5 required minor manual corrections along the entire TFS surface. Therefore, the actual speedup is probably much higher regarding automated post-processing or manual refinement steps necessary to fix atlas segmentation failures. The exact reason for the high rate of atlas mis-segmentations is unclear. It cannot be excluded that a better parameterization of the deformable registration could improve the success ratio. As mentioned, the very thin and, at times, low-contrast semi-circular canals would remain a challenge for atlas registration.

### 4.2. Comparison to Currently Available Neural Network Algorithms for MR Inner Ear Segmentation

In recent years, deep learning has revolutionized medical image analysis, particularly segmentation (73). Among an ever-growing number of architectures and approaches proposed for volumetric segmentation, two of the most popular and successful methods (74, 75) are 3D U-Net (49) and the previously proposed V-Net (48). In addition, the latest published suggestions for inner ear segmentation can also be seen in this development- whether for CT (76–78) or MRI (32, 33). In the following, currently available neural network algorithms used for MR inner ear (IE) segmentation will be compared (see Table 3 for an overview).

**Table 3:** Overview of MR IE deep learning segmentation algorithms in comparison.

	**IE-Vnet**	**IE-Unet**	**INHEARIT**
Machine learning technique	Deep learning	Deep learning	Deep learning
Network structure	3D Vnet (48)	3D Unet (49)	2D CNN based on VGG-19 (79)
Input	T2-weighted sequences	T2-weighted sequences	Hydrops-Mi2 (80)
Output	3D TFS segmentation	3D TFS segmentation	2D hydrops ratio
Output resolution	0.2 x 0.2 x 0.2 mm^3^	0.45 x 0.45 x 0.45 mm^3^	0.5 x 0.5 mm^2^
**(A) Training and testing parameters**		
Ground truth	Semi-manual atlas-based segmentation	Manual segmentation	Manual segmentation
**Training dataset**	**Mono-centric (n=179)**	**Mono-centric (n=944)**	**Mono-centric (n=124)**
*Features*	*3T, multi-scanner, multi-scale*	*1.5T, 3T, multi-vendor, multi-scale*	*3T, 1 scanner, 1 scale*
*Participants*	*Vestibular pathologies and HC*	*IE pathologies*	*MD, VM, VN*
**Test dataset**	**Mono-centric (n=80)**	**Multi-(n=3)-centric (n=276)**	***5-fold cross validation of***
*Features*	*3T, multi-scanner, multi-scale*	*1.5T, 3T, multi-vendor, multi-scale*	***Training dataset***
*Participants*	*Vestibular pathologies and HC*	*IE pathologies*	*see above*
**(B) Model performance**			
Accuracy (Dice)	0.90 ± 0.02	0.87 (CI 0.87-0.88)	0.83 ± 0.04
**Robustness**	**100% in test sets D2-5**	**98.3% in test centers B-D**	**n.r**.
*To artifacts*	*n.a*.	*Yes*	*n.r*.
*To outliers*	*Yes*	*Yes*	*n.r*.
*To noise*	*Yes*	*Yes*	*n.r*.
Speed (localization/segmentation)	25s / 0.19 s	n.r. / 6.5 s	n.r. / within 1 s
Ability to segment diseased IE	Yes	Yes	Yes
Full automatization	Yes	Yes	Yes
Manual intervention needed	IE localization	Data preparation	No
Data availability	No	No	No
Model availability	Yes	No	No
Source availability	Yes	No	No

*In the following the current study is referred to as “IE-Vnet.” The approach of Vaidyanathan et al. (33) is referred to as “IE-Unet.” Cho et al. (32) called their approach “INHEARIT” and are referred to as such. INHEARIT offers an automatic 2D area ELH (endolymphatic hydrops) ratio segmentation customized to the needs of a clinical radiologist, while IE-Vnet and IE-Unet enable 3D volumetric TFS segmentation with broad usability. The comparison considers a) parameters of the training and testing of the models, as well as their b) performance. IE-Vnet and Unet represent a similar approach to the same problem and can be complementary. However, while Unet offers a large dataset, IE-Vnet operates at a more than twice higher resolution, and its pre-trained model and accompanying codebase will be published open-source. CI, confidence interval 95%; ELH, Endolymphatic hydrops; HC, Healthy controls; Hydrops-Mi2, HYbriD of Reversed image Of Positive endolymph signal and native image of positive perilymph Signal- Multiplied with heavily T2-weighted MR cisternography; IDL, idiopathic hearing loss; IE, inner ear; INHEARIT, INner ear Hydrops Estimation via ARtificial InTelligence; MD, Morbus Mnire; MRC, MR cisternography; n.a., not analyzed; n.r., not reported; TFS, Total fluid space; VM, Vestibular migraine; VN, Vestibular neuritis*.

To the best of our knowledge, there are two machine learning MR IE segmentation proposals to date. First, Cho et al. (32) developed an automated measurement of 2D cochlea and vestibulum hydrops ratio from iMRI using CNN-based segmentation. Its primary difference is its usage of 2D data and focused usability on ELH area ratios in cochlea and vestibule. This tool should prove helpful to make ELH classifications (4, 5, 8, 9) more objective and comparable for clinical radiologists during the diagnostic assessment. For research purposes, ELH classification and 2D- or 3D- quantification methods were reliable and valuable for diagnosing endolymphatic hydrops (25). However, the reliability increases from ELH classification to 2D- and again to 3D-quantification methods (10). A model for complete 3D segmentation of TFS, including semi-circular canals (SCC), not only enables 3D volumetric analyses but gives it a substantially wider application area, e.g., IE surgical planning.

Second, Vaidyanathan et al. (33) recently suggested a fully automated segmentation of the inner ears TFS based on deep learning similar to our current approach. There are many overlaps in methodology and application, e.g., a similar network architecture. In the following, it will be referred to as IE-Unet. Compared to IE-Vnet, IE-Unet does not need to localize the inner ears in a separate pre-processing step. On the other hand, IE-Vnet operates at a more than twice higher resolution (0.2 mm isotropic vs. 0.45 mm), which leads to smoother surface boundaries of the output segmentation and can better deal with partial volume effects due to low voxel resolution in MRI. Notably, both solutions follow a similar approach to the same problem (IE MR TFS segmentation), which highlights their relevance and value compared to the method of Cho et al., whose usability is limited to the hydrops ratio in cochlea and vestibulum. Most importantly, though, both IE-Vnet and IE-Unet are highly complementary, making both trained models highly valuable. Therefore, we are choosing to publish our pre-trained model and accompanying code for training and inference open-source replication in other centers and alleviate similar studies in the community.

### 4.3. Clinical Implications

Deep learning models for medical image analysis have reached a maturity (74) that makes them relevant for further clinical and research-based investigations of the inner ear in the neuro-otological and vestibular domain. Once released, the proposed inner ear TFS segmentation approach using a dedicated deep learning (DL) model based on a V-Net architecture (IE-Vnet) has the potential to become a core tool for high-volume trans-institutional studies in vestibulocochlear research, such as on the endolymphatic hydrops (ELH).

IE-Vnet bridges the current gap existing for available automatic 3D ELS quantification methods. In particular, its input can be seamlessly combined with a previously published open-source pipeline for automatic iMRI ELS segmentation (27) via the TOMAAT module (81) in 3DSlicer (82).

### 4.4. Limitations and Future Work

There are methodical limitations in the current study that need to be considered in interpreting the data. One limitation of IE-Vnet in its current form is its reliance on a pre-localization and cropping of a cubical inner ear ROI obtained via deformable registration of the FHT and a transfer of the inner ear annotations. Their computational time was not considered in the discussion since both IE-Vnet, and the IET atlas-segmentation assume a previous localization and ROI cropping of the inner ear. The pre-processing steps are limited to localizing the left and right inner ear in the present work. This can be achieved fully automatically using a full-head registration and requires no manual interaction (other than, e.g., a post-registration visual inspection of whether the cropped ROI indeed contains the inner ear). In the current study, inner ear localization was successful for all 100% of inner ears. This can be achieved fully automatically using a full-head registration and requires no manual interaction (other than e.g., a post-registration visual inspection whether the cropped ROI indeed contains the inner ear). In this study, inner ear localization was successful for all 100% of inner ears. Given that IE-Vnet is trained to be robust toward a localization uncertainty of ~1 cm (cf. augmentations in Section 2.5.2) this registration can be parametrized at a reasonably low resolution (e.g., deformation fields at 5 mm resolution). Consequently, in our study, inner ear localization via deformable registration was comparatively fast and took 25 s for both inner ears of each subject on a commodity laptop with 4a CPU. Alternatively, the inner ears can also be manually localized using landmark annotation. Depending on the workstation hardware and registration parametrization, a fully automatic inner ear ROI localization could be performed in 1–2 min. A manual localization is much faster and requires two clicks, which can be performed in the order of seconds. However, it would be attractive to incorporate this step into the deep learning architecture itself, either via a cascaded setup of two networks (83), one for ROI localization and one for segmentation (IE-Vnet), or via a sliding-window inference approach (84). Both approaches are exciting avenues for future work. Another issue is that rare cases with strong artifacts can still lead to mis-segmentations (e.g., Figure 6D). However, such cases are statistically rare (long-tail problem) and challenging to solve. Instead, prior knowledge of the shape and topology of the inner ears TFS could be incorporated into the regularization model, e.g., through statistical shape models (85, 86).

## 5. Conclusion

The current work proposes a novel volumetric MR image segmentation approach for the inner ears total fluid space (TFS) using a dedicated deep learning (DL) model based on V-Net architecture (IE-Vnet). IE-Vnet demonstrated high accuracy, speedy prediction times, and robustness toward domain shifts. Furthermore, its output can be seamlessly combined with a previously published open-source pipeline for automatic iMRI ELS segmentation. Taken together, IE-Vnet has the potential to become a core tool for high-volume trans-institutional studies of the inner ear in vestibular research and will also be released as a free and open-source toolkit.

## Data Availability Statement

The original contributions presented in the study are included in the article's Supplementary Material, further inquiries can be directed to the corresponding authors.

## Ethics Statement

The studies involving human participants were reviewed and approved by Ethics Commission of the medical faculty of the Ludwig-Maximilians-Universität, Munich, Germany. The patients/participants provided their written informed consent to participate in this study.

## Author Contributions

S-AA and JF: conception, design of the study, analysis of the data, drafting the manuscript, and providing funding. GV: acquisition and analysis of the data. MD: conception and design of the study, drafting the manuscript, and providing funding. VK: conception, design of the study, acquisition, analysis of the data, drafting the manuscript, and providing funding. All authors contributed to the article and approved the submitted version.

## Funding

This work was partially funded by the German Foundation of Neurology (Deutsche Stiftung Neurologie, DSN), Verein zur Förderung von Wissenschaft und Forschung an der Medizinischen Fakultät der LMU (Association for the Promotion of Science and Research at the LMU Medical Faculty), and the German Federal Ministry of Education and Research (BMBF) via the German Center for Vertigo and Balance Disorders (DSGZ, Grant No. 01 EO 0901).

## Conflict of Interest

S-AA was employed by NVIDIA GmbH. The remaining authors declare that the research was conducted in the absence of any commercial orfinancial relationships that could be construed as a potential conflict of interest.

## Publisher's Note

All claims expressed in this article are solely those of the authors and do not necessarily represent those of their affiliated organizations, or those of the publisher, the editors and the reviewers. Any product that may be evaluated in this article, or claim that may be made by its manufacturer, is not guaranteed or endorsed by the publisher.

## Footnotes

^1^ANTs open-source code and binaries: https://stnava.github.io/ANTs/.

^2^3D Slicer open-source code and binaries: https://www.slicer.org/.

^3^Project MONAI documentation and code: https://monai.io/.

## Supplementary Material

### Datasets D1-5: Measurement of the Auditory, Semicircular Canal, and Otolith Functions

Diagnostic work-up included a careful neurological (e.g., history-taking, clinical examination), and neuro-orthoptic assessment with, e.g., Frenzel goggles, fundus photography, adjustments of the subjective visual vertical (SVV), video-oculography (VOG) during caloric stimulation and head-impulse test (HIT), as well as pure tone audiometry (PTA). A tilt of the SVV is a sensitive sign of a graviceptive vestibular tone imbalance. SVV was assessed when sitting in an upright position in front of a half-spherical dome with the head fixed on a chin rest (87). A mean deviation of > 2.5 from the true vertical was considered a pathological tilt of SVV (87). The impairment of vestibulo-ocular reflex (VOR) in higher frequencies was measured by using high-frame-rate VOG with EyeSeeCam ((88), EyeSeeTech, Munich, Germany). A median gain during head impulses <0.6 (eye velocity in °/s divided by head velocity in °/s) was considered a pathological VOR (89). Furthermore, horizontal semicircular canal responsiveness in lower frequencies was assessed by caloric testing with VOG. This was done for both ears with 30°C cold and 44° C warm water. Vestibular paresis was defined as >25% asymmetry between the right- and left-sided responses (90).

## Abbreviations

±: Standard deviation
2D: Two-dimensional
3D: Three-dimensional
ANTS: Advanced Normalization Toolkit
aSCC: anterior semi-circular canal
D1, Dataset 1: Training dataset
D2, Dataset 2: Test dataset
D3, Dataset 3: Test dataset
D4, Dataset 4: Test dataset
D5, Dataset 5: Test dataset
DL: Deep learning
ELH: Endolymphatic hydrops
ELS: Endolymphatic space
FLAIR: Fluid-attenuated inversion recovery
FH: Full-head
FHT: Full-head template
FOV: Field-of-view
GBCA: Gadolinium-based contrast agent
Gd: Gadolinium
GRAPPA: Generalized auto-calibrating partially parallel acquisition
HC: Healthy control
hSCC: horizontal semi-circular canal
IET: Inner ear template
IE-Vnet: Inner ear dedicated deep learning model based on a V-Net architecture
iMRI: Delayed intravenous gadolinium-enhanced MRI of the inner ear
iv: Intravenous
L: Left
MRI: Magnetic resonance imaging
n: Number
OTB: Optimal Template Building
PLS: Perlymphatic space
pSCC: posterior semi-circular canal
QC: Quality-control (led)
R: Right
ROI: Region-of-interest
SCC: Semi-circular canal
SQ: Semi-quantitative
SPACE: Sampling perfection with application-optimized contrasts by using different flip angle evolutions
SyN: Symmetric Normalization
TFS: Total fluid space.
