# Supplementary material for: IE-Vnet: Deep Learning-Based Segmentation of the Inner Ear's Total Fluid Space
Source: Front Neurol. 2022 May 11;13:663200. doi: 10.3389/fneur.2022.663200 (PMC9130477; doi:10.3389/fneur.2022.663200)
Supplement: Supplementary file 1 [file Data_Sheet_1.pdf]

Supplementary Material

DATASETS D1-5: MEASUREMENT OF THE AUDITORY, SEMICIRCULAR CANAL, AND OTOLITH FUNCTIONS

Diagnostic work-up included a careful neurological (e.g., history-taking, clinical examination), and neuro-orthoptic assessment with, e.g., Frenzel goggles, fundus photography, adjustments of the subjective visual vertical (SVV), video-oculography (VOG) during caloric stimulation and head-impulse test (HIT), as well as pure tone audiometry (PTA). A tilt of the SVV is a sensitive sign of a graviceptive vestibular tone imbalance. SVV was assessed when sitting in an upright position in front of a half-spherical dome with the head fixed on a chin rest [Dieterich and Brandt (1993)]. A mean deviation of > 2.5 from the true vertical was considered a pathological tilt of SVV [Dieterich and Brandt (1993)]. The impairment of vestibulo-ocular reflex (VOR) in higher frequencies was measured by HIT (34) using high-frame-rate VOG with EyeSeeCam [Schneider et al. (2009), EyeSeeTech, Munich, Germany]. A median gain during head impulses < 0.6 (eye velocity in °/s divided by head velocity in °/s) was considered a pathological VOR Halmagyi and Curthoys (1988). Furthermore, horizontal semicircular canal responsiveness in lower frequencies was assessed by caloric testing with VOG. This was done for both ears with 30°C cold and 44°C warm water. Vestibular paresis was defined as $>25\%$ asymmetry between the right- and left-sided responses [Jongkees et al. (1962)].

REFERENCES

Dieterich, M. and Brandt, T. (1993). Ocular torsion and tilt of subjective visual vertical are sensitive brainstem signs. *Annals of Neurology* 33, 292–299. doi:10.1002/ana.410330311

Halmagyi, G. M. and Curthoys, I. S. (1988). A clinical sign of canal paresis. *Archives of Neurology* 45, 737–739. doi:10.1001/archneur.1988.00520310043015

Jongkees, L. B., Maas, J. P., and Philipszoon, A. J. (1962). Clinical nystagmography. A detailed study of electro-nystagmography in 341 patients with vertigo. *Practica Oto-Rhino-Laryngologica* 24, 65–93

Schneider, E., Villgrattner, T., Vockeroth, J., Bartl, K., Kohlbecher, S., Bardins, S., et al. (2009). EyeSeeCam: an eye movement-driven head camera for the examination of natural visual exploration. *Annals of the New York Academy of Sciences* 1164, 461–467. doi:10.1111/j.1749-6632.2009.03858.x
